# A microsurgical technique for catheter insertion in the rat femoral artery^1^

**DOI:** 10.1590/s0102-865020200100000004

**Published:** 2020-11-23

**Authors:** Kazuhisa Sugai, Yoji Hakamata, Tomoyoshi Tamura, Masaharu Kataoka, Masahiko Fujisawa, Motoaki Sano, Eiji Kobayashi

**Affiliations:** I Master, Division of Basic Science, School of Veterinary Nursing and Technology, Faculty of Veterinary Science, Nippon Veterinary and Life Science University, Tokyo, Japan. Conception and design of the study, analysis and interpretation of data, manuscript writing.; II DVM, PhD, Division of Basic Science, School of Veterinary Nursing and Technology, Faculty of Veterinary and Research Center for Animal Life Science, Nippon Veterinary and Life Science University, Tokyo, Japan. Conception and design of the study, analysis and interpretation of data, manuscript writing.; III MD, PhD, Emergency and Critical Care Medicine, Keio University School of Medicine, Tokyo, Japan. Substantive scientific and intellectual contributions to the study, conception and design.; IV MD, PhD, Department of Cardiology, Keio University School of Medicine, Tokyo, Japan. Substantive scientific and intellectual contributions to the study, conception and design.; V MD, PhD, Department of Organ Fabrication, Keio University School of Medicine, Tokyo, Japan. Conception and design, technical procedures, manuscript preparation, final approval.

**Keywords:** Catheterization, Microsurgery, Telemetry, Vital Signs, Rats

## Abstract

**Purpose:**

To modify a surgical catheterization method using the bent needle introducer in small animals.

**Methods:**

Eight-week-old male Lewis rats were used in the study. A needle introducer was created by bending a 21G injection needle at 45°. The bent needle introducer was used for catheter insertion into the left femoral artery of the rats under anesthesia. As a control, a catheter was directly inserted into the blood vessel without the introducer. The insertion time of each method was measured. Blood pressure and heart rate were measured 24 h after catheter insertion using the telemetry system.

**Results:**

Using the introducer, the catheter was successfully inserted within a short time in all rats. Without the introducer, a longer duration was required for catheter insertion. The frequency of the insertion with no catheter-based errors with the introducer tended to be higher than that without the introducer. The mean arterial pressure and heart rate 24 h after catheter insertion in each group were almost the same.

**Conclusions:**

We developed a surgical catheterization method using the introducer in small animals. This could potentially reduce the frequency of the insertion with catheter-based errors and insertion time.

## Introduction

Monitoring the vital signs (e.g., blood pressure, heart rate and electrocardiogram) in safety pharmacology studies using rodents (e.g., rats and mice) under unanesthetized and unconstrained conditions is essential to determine the effectiveness and side effects of the drugs^1^ ^-^ ^4^.

Telemetry systems are widely used for collecting the physiologic parameters (e.g., blood pressure and heart rate) from the awake and freely moving small animals as stabilized data^5^. For operating such telemetry systems, the surgeons must possess microsurgical expertise to insert a catheter in the blood vessels of a small animal and to maintain this system over time. Various catheter insertion methods have been developed depending on the objective of parameter measurement objectives^6^ ^-^ ^8^. Catheter-specific forceps and catheter introducers are also available for aiding catheter insertion.

We believe that microsurgical expertise can minimize surgical errors when working with experimental animals; thus, small animal models with fewer individual variations can be created. Consequently, we have focused on disseminating these techniques^9^ ^-^ ^11^. This study aimed to analyze the protocol for simplifying the insertion of a catheter into a blood vessel using a bent needle introducer, a commercially available injection needle whose tip has a gradual curve.

## Methods

The 8-week-old male Lewis rats used in the study were purchased from Charles River Laboratories, Japan (Kanagawa, Japan), acclimatized for 1 week, and used for experiments when they were 9 weeks of age. The animals were bred in an environment with room temperature of 24±1°C, humidity of 50±10%, and a 14 h:10 h light-to-dark cycle. They were allowed free access to commercially-available solid feed (EF, Oriental Yeast Co., Ltd., Tokyo, Japan) and water. Rats were randomly divided into two groups (with-introducer group: N=7, without-introducer group: N=6). The study was approved by the Institutional Animal Care and Use Committee (Nippon Veterinary and Life Science University, Tokyo, Japan. Permit No. 2019K-10), which works in accordance with Nippon Veterinary and Life Science University guidelines for the care and use of laboratory animals. All sections of this report adhere to the ARRIVE Guidelines for reporting animal research^12^. A completed ARRIVE checklist is included in S1 Table. All surgeries were performed under general anesthesia using isoflurane (Mylan Inc., Osaka, Japan). All efforts were made to minimize suffering.

### Instruments

The catheters used in the experiments were telemetric transmitters for automatic measurement systems (HD-S10, Physio Tel® HD Telemetry, Data Science International, Minneapolis, MN, USA). The transmitter body consists of an electronic module, sensor and battery. A catheter filled with fluid is connected to the transmitter body. The catheter tip is filled with gel, which prevents blood flow into the catheter and thrombogenesis. The sterilized catheters were soaked in sterilized physiologic saline for 15 min before insertion to prevent thrombogenesis.

**Figure 1:**
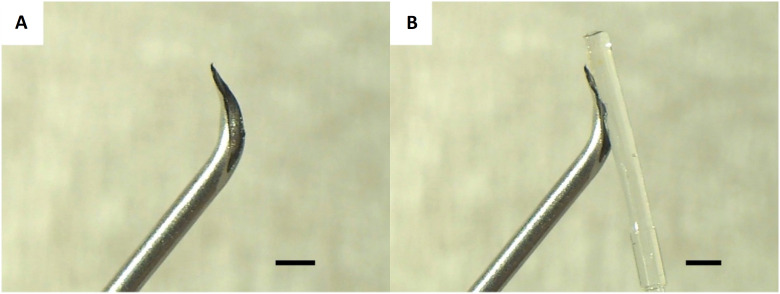
Bent needle introducer. **(A)** The injection needle (21G) was bent by 45° so that the edge surface faces outwards. **(B)** The telemetry catheter is inserted into the blood vessel while following the groove of the bent injection needle. Scale bar: 1mm.

An injection needle (21GX1/2 RB, outer diameter: 2.5 Fr, FLOMAX, Nipro, Osaka, Japan) was used as the bent needle introducer to aid with the insertion of the catheter tip (outer diameter: 2 Fr) into the artery. The needle tip was bent to an angle of 45° with forceps so that the edge surface faced outwards (Fig. 1A). The catheter was inserted into the blood vessel as it followed the groove of the bent injection needle (Fig. 1B).

### Preparation for insertion of the telemetry catheter

**Figure 2:**
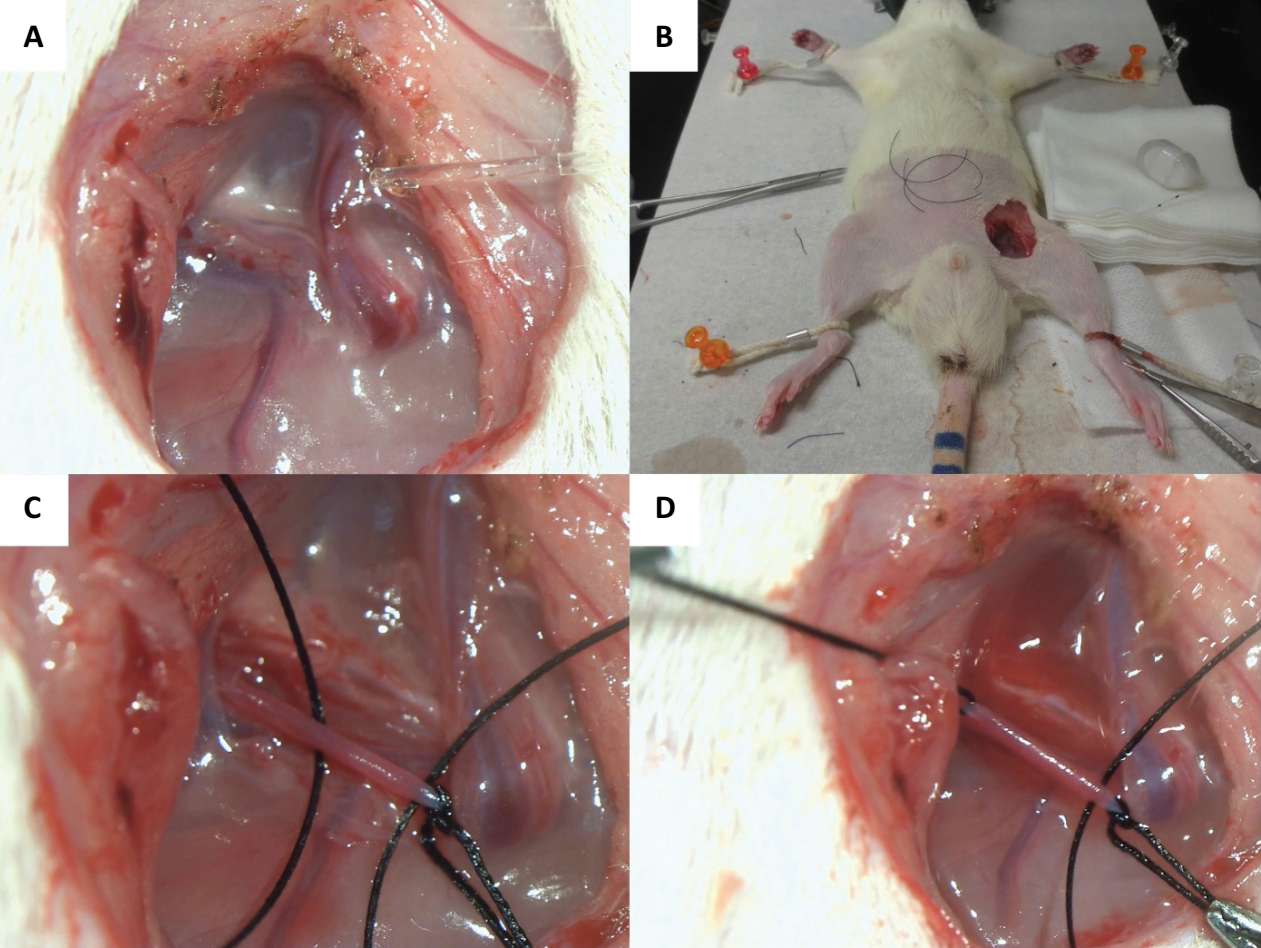
Preparation for the insertion of the telemetry catheter in the rat femoral artery. **(A)** After making an incision in the left inguinal region, the subcutaneous tissue is retracted, and the left femoral artery and femoral nerve are exposed. **(B)** Three 4-0 silk threads used for ligation are placed in the operating field, and the telemetry catheter to be inserted is placed on the left abdominal side. **(C)** After the femoral artery is separated, the peripheral side is ligated with a 4-0 silk thread and tightened with forceps. Two of the same threads are passed through the heart side as well. **(D)** A small gauze with 1% xylocaine is left to stand for 30 s in this area to prevent blood vessel contraction, following which the silk threads on the heart side are tightened with forceps.

The rats were anesthetized by inhalation of 2% isoflurane. During surgery, the body temperature of all animals was maintained using a heating pad (KN-475-3-40, Natsume, Tokyo, Japan). Their four limbs were constrained in the dorsal position with a rope, and the skin in the operating field was disinfected with 70% ethanol gauze. An approximately 2 cm incision was made in the left inguinal region parallel to the rectus abdominis muscle. The subcutaneous tissue was retracted, and the left femoral artery and femoral nerve were exposed 2 cm from the abdominal wall (Fig. 2A). Three 4-0 silk threads used for ligation were placed in the operating field; telemetry catheter to be inserted was placed in the left abdominal side, and the catheter tip was set up along the left femoral artery (Fig. 2B).

The femoral artery was carefully separated using the microscale forceps under a microscope (SZX-7, OLYMPUS, Tokyo, Japan). The peripheries were ligated with 4-0 silk thread and tightened with forceps. Two of the same silk threads were passed through the heart side as well (Fig. 2C). A small gauze with 1% xylocaine was left to stand for 30 seconds in this area to prevent blood vessel contraction, following which the silk threads on the heart side were tightened with forceps (Fig. 2D).

### Insertion of the telemetry catheter

**Figure 3:**
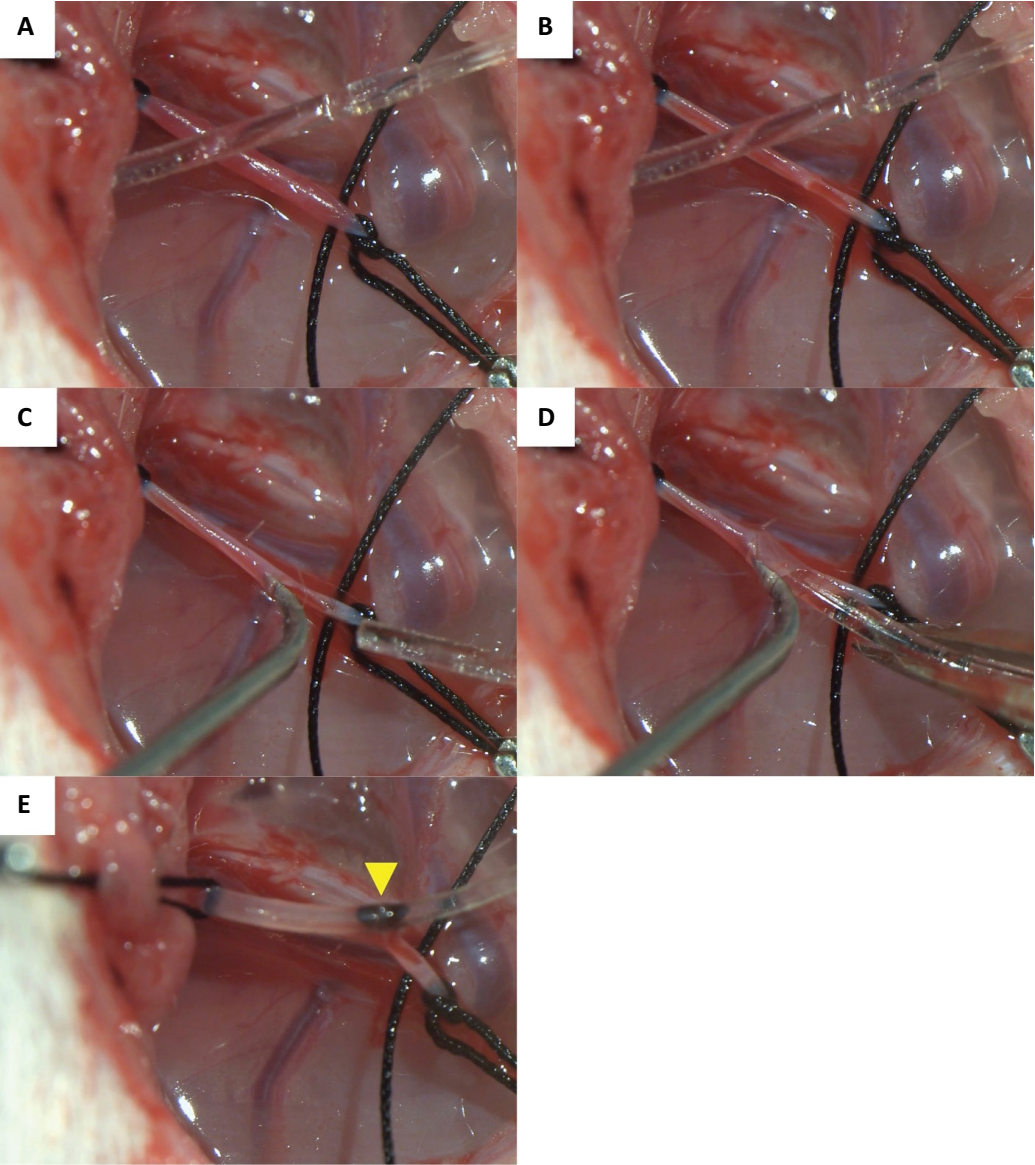
insertion of the telemetry catheter in the rat femoral artery. **(A)** Before making a semi-circular incision on the anterior wall in the femoral artery. **(B)** After making a semi-circular cut in the anterior wall of the femoral artery. **(C)** The tip of the bent needle introducer is inserted into the blood vessel from the cut blood vessel side, and the blood vessel opening is expanded. **(D)** The tip of the catheter is advanced along the groove of the injection needle and inserted into the blood vessel. **(E)** The catheter is left in place once it has advanced approximately 5 cm towards the heart. The arrows indicate the position 5 cm from the tip of the catheter.

**Figure 4:**
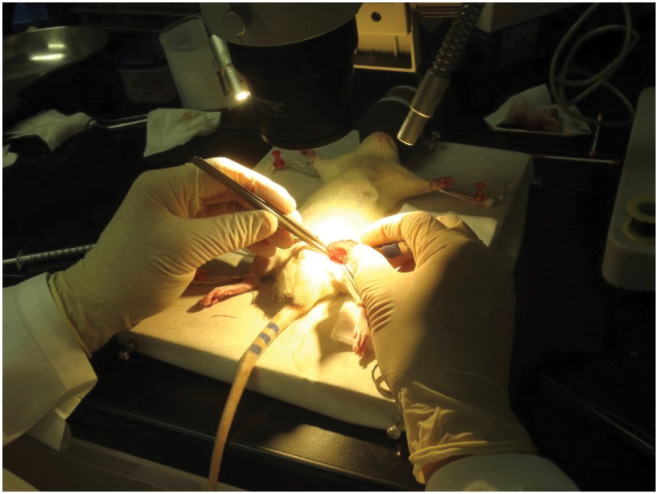
Position of the hands on the operating table. The lower parts of both palms are placed on the operating table to prevent shaking of the hands.

A semi-circular opening in the anterior wall of the separated femoral artery was created using microscale scissors (Fig. 3A, B). A thread on the heart side was pulled, and the hemorrhaging was stopped from the cut side. The tip of the previously-prepared bent needle introducer was inserted into the blood vessel from the cut surface and slightly elevated, following which the opening of the blood vessel was expanded (Fig. 3C). The tip of the catheter was advanced along the groove of the injection needle and inserted into the blood vessel (Fig. 3D). Following confirmation of catheter insertion in the artery, the tightened thread was loosened, and the catheter was further advanced 5 cm towards the heart side and left in place once it was positioned in front of the site of initiation of the left renal artery (Fig. 3E). The inserted catheter was firmly fixed in place using two threads on the heart side and one thread on the peripheral side to prevent its extraction. The lower parts of both palms were placed on top of the operating table to prevent shaking of the hands, which is important when performing experimental microsurgical techniques (Fig. 4).

As a control, catheters were directly inserted into the blood vessel of rats using standard microscale forceps without the aid of the bent needle introducer. Following the implantation of the catheter, the transmitter body was inserted into a subcutaneous pocket created on the left lower back, and the skin was sutured. After surgery, all animals received subcutaneous administration of antibiotic cefmetazole sodium (1 mL/kg, cefmetazole sodium for intravenous, 0.1 g/mL, Nipro, Osaka, Japan) to dorsal of the neck, and were monitored until fully conscious. The rats were individually housed in separate cages, and the condition of the wound was observed until the measurement of blood pressure and heart rate.

The time taken for catheter insertion in each method was measured. The insertion time was defined as the time from making an incision on the anterior wall of the femoral artery (Fig. 3B) to leaving the catheter in place once it advanced approximately 5 cm toward the heart side (Fig. 3E).

### Blood pressure and heart rate measurement

The measurement of the blood pressure and heart rate was initiated 24 h after catheter insertion; the blood pressure and heart rate were measured for 30 min using the telemetry system software (Ponemah Ver. 6.3, Data Science International). The data recorded in the first 5 min were excluded, and valid data for the first 1 min of each 5-minute block were used. Measurement was performed by M.F. who was blinded to group allocation.

### Statistical analysis

All data are presented as mean ± standard deviation. All statistical analyses were conducted using EZR (Saitama Medical Center, Jichi Medical University, Saitama, Japan)^13^. EZR is a graphical user interface for R (The R Foundation for Statistical Computing, Vienna, Austria). Comparisons were performed using the Student's T-test, Welch's T-test, or Fisher's exact test, as appropriate. A p-value <0.05 was considered statistically significant.

## Results

### Advantage of the introducer and the insertion time

**Figure 5:**
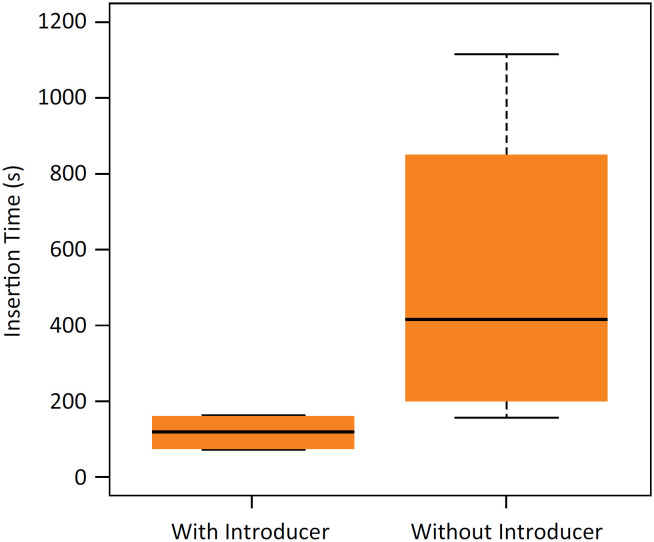
Time taken for catheter insertion in each method. The insertion time in the with-introducer group tended to be shorter than that in the without-introducer group, although there were no significant differences between the two groups (p=0.158, Welch´s T-test). N=4 in the with-introducer group, N=4 in the without-introducer group.

Using the bent needle introducer, the catheter was successfully inserted within a short time in all rats. However, on using the insertion method wherein the bent needle introducer was not used, there was difficulty in catheter insertion, gel leakage at the catheter tip was observed, and gel was refilled; thus, a longer duration was required for insertion. The insertion time ranged from 74 to 165 seconds for the with-introducer group and from 155 to 1109 seconds for the without-introducer group. The average insertion time was 119.0±44.5 and 523.3±374.6 seconds for the with- and without-introducer groups, respectively. The insertion time tended to be shorter in the with-introducer group than in the without-introducer group, although there were no significant differences in the insertion time between the two groups (p=0.158; Fig. 5).

Gel leakage at the catheter tip was regarded as a catheter-based error, and the frequency of the insertion with errors between the two groups was analyzed by Fisher's exact test. The frequency of the insertion with no catheter-based errors tended to be higher in the with-introducer group, although there were no significant differences between the two groups (p=0.143, Table 1).

**Table 1:** Frequency of insertion with catheter-based errors for each method.

Group	Insertion time	Frequency of insertion with errors (N)	Frequency of insertion with no errors (N)	Fisher's exact p value
with introducer	74, 77, 160, 165	0	4	0.143
without introducer	155, 243, 586, 1109	3	1	

The frequency of insertion with no errors tended to be higher in the with-introducer group than in the without-introducer group (p<0.05*).

### Blood pressure and heart rate

**Figure 6:**
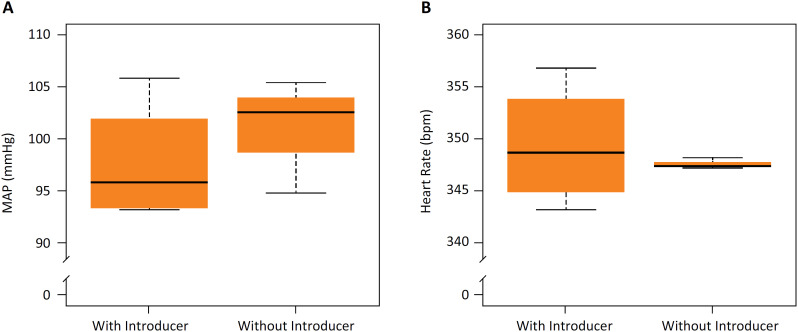
Mean arterial pressure and heart rate 24 h after catheter insertion. **(A)** No significant differences were observed in the mean arterial pressure between the two groups 24 h after the surgery (p=0.492, Student´s T-test). **(B)** No significant differences were observed in the surgery (p=0.605, Welch´s T-test). N=4 in the with-introducer group, N=3 in the without-introducer group. MAP: mean arterial pressure.

The mean arterial pressure 24 h after catheter insertion was 97.7±5.1 mmHg for the with-introducer group and 100.9±4.5 mmHg for the without-introducer group, and no significant differences were observed between the two groups (p=0.492; Fig. 6A). The heart rate was 349.3±5.1 bpm in the with-introducer group and 347.6±0.4 bpm in the without-introducer group, and no significant differences were observed between the two groups (p=0.605; Fig. 6B).

## Discussion

The techniques used to insert telemetry catheters into the blood vessels of small rodents have been modified several times to date, according to the telemetry parameter types or objectives^6^ ^-^ ^8^. In this study, we developed a safe, yet secure, method for inserting a catheter into the blood vessels of rats using a self-made bent needle introducer. A method similar to our method was previously used for catheter insertion in a large-sized experimental cat model^14^. This method is considered extremely effective; however, its use is not widespread in small animals, such as rats or mice. Moreover, at the time this study was conducted, we were unaware of the study conducted by Miller *et al.* ^14^ and came across their work while reporting on these results. A noteworthy aspect of the independent design of our introducer was that we used the groove on the cut surface of a commercially available injection needle, which is quite common in the laboratory, in order to guide the catheter insertion. Although catheter-specific forceps that prevent catheter deformation and catheter introducers are available, the present method allows for easy catheter insertion into the blood vessel by sliding it down the groove of the curved injection needle. This method applies the general principles of inserting a catheter into a blood vessel while following the inner needle of the intravenous catheter. In addition, the bent needle introducer developed in this study is very economical and easy to create, and different types of needles can be used depending on the thickness of the blood vessel and catheter.

In this study, there were no significant differences in the insertion time between the two groups, although there was a tendency for the insertion time to be shorter in the with-introducer group than in the without-introducer group. However, we also showed that the frequency of the insertion with no catheter-based errors was significantly higher in the with-introducer group than in the without-introducer group, although there were no significant differences between the two groups. These results suggested that the reduction of catheter-based errors could shorten the insertion time. In addition, since gel leakage was noticed and refilled, blood pressure and heart rate could be measured, and there were no significant differences between the two groups, although if gel leakage had not been noticed, the accurate measurement would have been impossible, or telemetry transmitter would have been broken at worst.

Using our proposed method, even relatively inexperienced surgeons will be able to easily insert a catheter into a narrow blood vessel. This study showed the effectiveness of the bent needle introducer for catheter insertion into the arteries of small animals; however, this technique may be used for all tubular structures, such as in the blood and lymphatic vessels, trachea or the urinary tract. Furthermore, this method markedly reduces the surgical time and is useful with respect to animal welfare.

## Conclusion

We developed and described in detail an easy and reliable protocol for telemetry catheter insertion into the blood vessel of a small rodent using a self-made bent needle introducer.
